# Simulation of Charged Particle Trajectories in the Neutron Decay Correlation Experiment abBA

**DOI:** 10.6028/jres.110.068

**Published:** 2005-08-01

**Authors:** Dharmin Desai, Geoffrey Greene, Rob Mahurin, David Bowman, John Calarco

**Affiliations:** University of Tennessee, Knoxville, TN 37996; Oak Ridge National Laboratory, Oak Ridge, TN 37831; Los Alamos National Laboratory, Los Alamos, NM 87545; University of New Hampshire, Durham, NH 03824

**Keywords:** abBA experiment, abBA spectrometer, charged particle trajectory, coincidence experiment, computer simulation, guiding center approximation

## Abstract

The proposed neutron decay correlation experiment, abBA, will directly detect the direction of emission of decay protons and electrons as well as providing spectroscopic information for both particles. In order to provide this information, the abBA experiment incorporates spatially varying electric and magnetic fields. We report on detailed simulations of the decay particle trajectories in order to assess the impact of various systematic effects on the experimental observables. These include among others; adiabaticity of particle orbits, tracking of orbits, reversal of low energy protons due to inhomogeneous electric field, and accuracy of proton time of flight measurements. Several simulation methods were used including commercial software (Simion), custom software, as well as analytical tools based on the use of adiabatic invariants. Our results indicate that the proposed field geometry of the abBA spectrometer will be substantially immune to most systematic effects and that transport calculations using adiabatic invariants agree well with solution of the full equations of motion.

## 1. Introduction

The goal of the neutron decay experiment abBA is to accurately measure the four T-even neutron beta decay correlation coefficients: *a, b, A*, and *B*. Since the Standard Model (SM) provides a detailed prediction for these coefficients, the precision measurement of *a, b, A*, and *B* provides a test of the SM and could provide evidence of new physics beyond the SM. In abBA, all of the coefficient measurements will be performed within the same experimental apparatus, which will allow for multiple crosschecks of possible systematic effects.

**Fig. 1:**
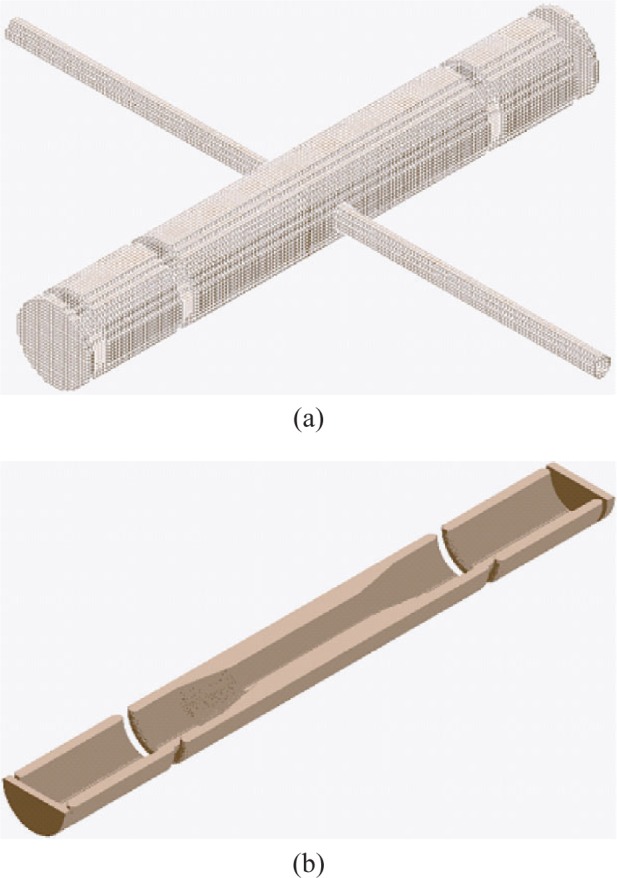
(a) is the 3D schematic of the abBA spectrometer, (b) is a 3D cross-sectional view of the spectrometer.

Decay protons emitted from a neutron beta decay have a maximum kinetic energy of ≈750 eV. In order to detect decay protons above the electronic noise of a Si detector, they must be accelerated to at least a few tens of keV. In the proposed abBA spectrometer (Figs. 1a, 1b), this acceleration of decay protons can be accomplished by maintaining a potential difference between the neutron decay volume and the Si detectors. Three cylindrically symmetric electrodes maintain the electric fields of the spectrometer with the central electrode at ≈30 kV and the end electrodes at ground.

**Fig. 2:**
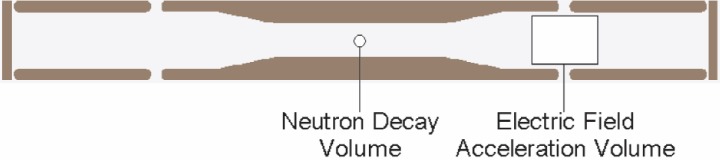
2D schematic of the abBA spectrometer showing location of the neutron decay volume and the electric field acceleration volume.

The electric fields in the abBA spectrometer are required to meet two principle design criteria: 1. Uniformity of electric field over the decay volume and 2. Reliable tracking of particle orbits in the acceleration volume (Fig. 2). A series of charged particle transport simulations were performed to validate the prototype’s geometry. The simulations used commercial software (SIMION 3D v7.0 1) and custom software.

## 2. Simulation Methods

SIMION 3D calculates the charged particle’s trajectory using a time-adaptive, 4th order Runge-Kutta algorithm to solve the equation of motion in three dimensions.

To run a virtual abBA experiment, the simulation software needs the capability to transport electrons and protons into the Si detectors to determine how many charged particles were reflected back into the spectrometer and needs the ability to save a large dataset, approximately 100 million events, in an SQL relational database for efficient and transparent data analysis. Custom software was developed to meet the above requirements in a fast, scalable, and flexible manner.

The custom software uses equations of motion derived from the guiding center approximation, combined with adiabatic invariance of magnetic flux through a particle orbit and conservation of energy, to calculate the charged particle’s trajectory Eqs. (1–3). In Eqs. (1–3) *R* is the centroid position of the particle’s orbit, *P*_‖_ is the momentum parallel to the magnetic field, ***B*** is the magnetic field, *c* is the speed of light, ***E*** is the electric field, *P*_⊥_ is the momentum perpendicular to the magnetic field, *t* is the time, *m* is the rest mass of the charged particle, γ*m* is the relativistic mass of the charged particle, and *q* is the charge of the charged particle. This method is accurate when two conditions are met: 1. Radius of gyration is small compared to the distance over which the electric and magnetic fields change appreciably, and 2. Electric force is small compared to the magnetic force.

$$\frac{dR}{dt}=\frac{P_{\|}}{\tilde{a}m}\frac{B}{B}+c\frac{E\times B}{B•B}+c\frac{P_{⊥}^{2}}{2\tilde{a}m}\frac{B\times \nabla B}{qB^{3}}$$ (1)

$$\frac{dP_{\|}}{dt}=q\frac{E\cdot B}{B}-\frac{P_{⊥}^{2}}{2\tilde{a}mB^{3}}B(B\cdot \nabla )B$$ (2)

$$\frac{d\tilde{a}}{dt}=q\frac{P_{\|}}{\tilde{a}m^{2}}\frac{E\cdot B}{B}$$ (3)

The guiding center approximation used in the custom software is based on the fact that the particle’s center of gyration moves approximately along the magnetic field line. To minimize error in the approximation, the custom software uses the deviation of the guiding center derived from the magnetic field line pertubatively.

## 3. Results

The symmetry of the abBA spectrometer implies that the highest potential lies at the mid-plane of the central electrode. Without sufficient longitudinal momentum, decay protons created on either side of the mid-plane will not overcome the potential barrier and will be reversed. These reversed protons must be considered a possible systematic effect in the measurement of the correlation coefficient *a* (*a* ≈ 0.1). To determine δ*a/a* ≈ 10^−3^, δ*a* must be approximately 10^−4^, which means that the number of reversed protons must be less than 1 in 10 000. A simple scaling argument concludes that the change in potential in the neutron decay volume should be no more than 2 µV to meet this condition. Such a condition is difficult to obtain using real conductors.

**Fig. 3:**
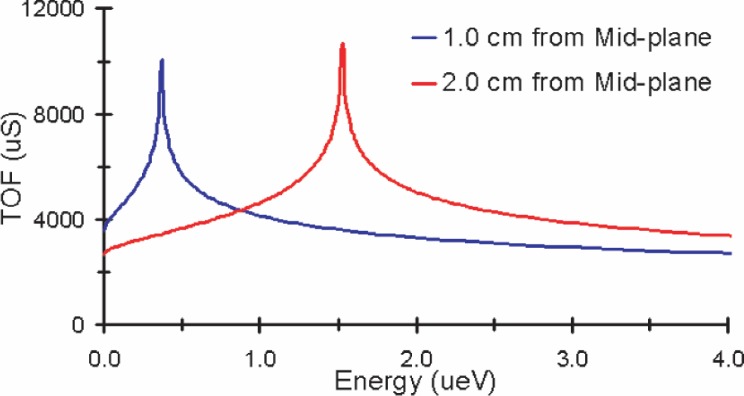
TOF plots for a simulated proton with energy 0 µeV to 4.0 µeV created on the central axis of the spectrometer. Initially, the proton is traveling in the direction of increasing potential and has velocity in the direction of the central axis.

Figure 3 shows that the energy corresponding to the peak of the asymptote is the minimum energy needed to overcome the potential barrier between the mid-plane and the location at which the decay proton is created. A proton with energy less than the asymptote energy is reversed and strikes the “wrong” detector, whereas a proton with energy greater than the minimum energy overcomes the potential barrier and strikes the “correct” detector. For example, the curve in Fig. 3 for a proton created 2.0 cm from the mid-plane shows an asymptotic energy of 1.6 µeV. This asymptotic energy of 1.6 µeV also represents the voltage drop of 1.6 µV between the mid-plane and the point 2.0 cm away.

More importantly, the experimental method, as a coincidence experiment, only counts events in which a proton is detected within a finite time window following the detection of an electron. Proton TOFs longer than the set time window are not recorded by the DAQ. As seen in Fig. 5, the time of flights for protons that undergo a reversal are much longer than the coincidence time window of approximately 100 µs; therefore, for the selected spectrometer geometry, no reversed protons are detected.

The proposed method assumes that the guiding center of the cyclotron orbit of any decay particle trajectory closely follows the magnetic field lines. This condition is met if no electric field is present. However, the addition of an electric field with a component perpendicular to the magnetic field causes the guiding center to drift. This drift is small if the magnetic forces dominate the electrostatic forces. In the acceleration region between the electrodes, the electric field is most inhomogeneous, and the magnetic field is uniform and only in the *z*-direction.

**Fig. 4:**
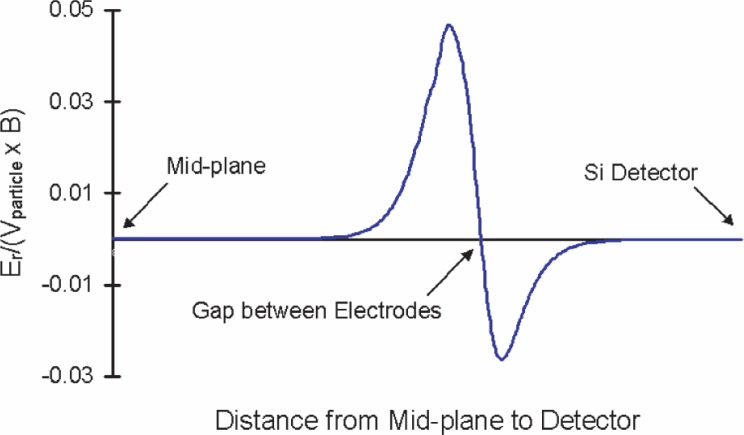
*E*_r_/(*V*_particle_ *B*) vs distance for a simulated off-axis proton. The trajectory of the proton goes through *E*_r_ maximum.

To insure that the magnetic forces dominate, the spectrometer design should satisfy the criteria that at all points in a charged particle’s trajectory the particle velocity *V*_particle_ ≫ *V*_EB_, where *V*_EB_ = E_r_/*B* and *E*_r_ is the radial component of the electric field. A rough scaling argument indicates that *V*_particle_ ≈ (50 – 100)*V*_EB_. This condition is verified by direct calculation of fields and particle velocities at each point in the trajectory. As seen in Fig. 4, the condition *V*_particle_ ≫ *V*_EB_ is well satisfied everywhere along the particle trajectory.

**Fig. 5:**
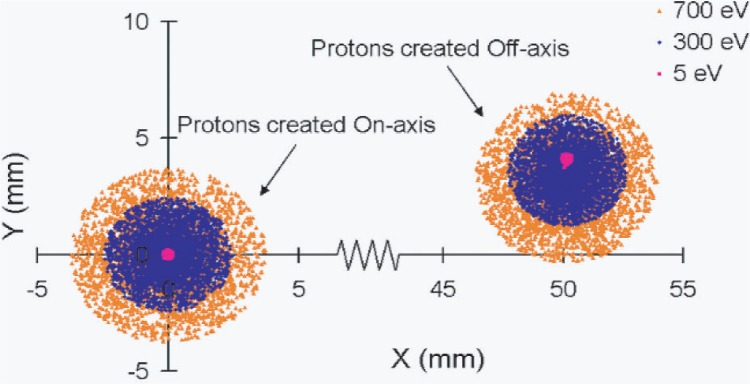
Final position distributions of simulated protons created on the mid-plane of the spectrometer. On-axis protons are created on the central axis, and off-axis protons are created 25 cm from the central axis.

A more detailed way of demonstrating that the “tracking condition” is met is by calculating the particle trajectories and showing that all particles reach the detector close to the point at which the magnetic field lines on which they started intersect the detector. Figure 5 shows the arrival locations of simulated protons with energies of 5 eV, 300 eV, and 700 eV emitted at random angles in a cone of 0° to 89°. Each distribution represents a collection of 10 000 protons. The distributions clustered around the origin are protons created in the mid-plane directly on the central axis, whereas the distributions that are clustered at approximately (50, 3) are created in the mid-plane 25 cm from the central axis. Due to the magnetic field expansion, the arrival locations of the protons are shifted in the *x*-direction with respect to the initial positions, and, in addition, the magnetic force creates a small shift in the *y*-direction. These simulations show that the spectro-meter design satisfies the field homogeneity and particle tracking criteria.

**Fig. 6:**
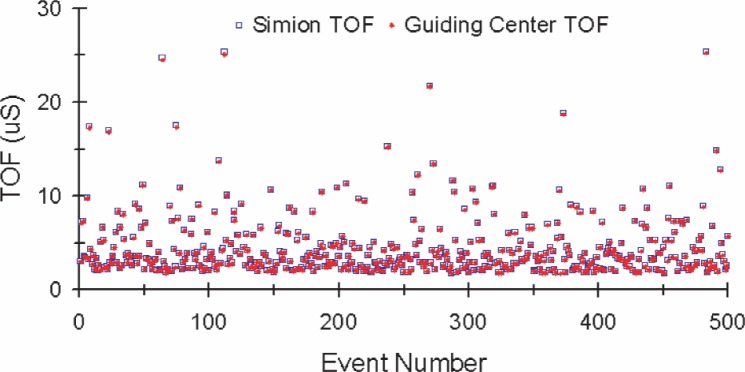
Proton TOFs generated by SIMION compared to proton guiding center TOFs generated by the custom software.

**Fig. 7:**
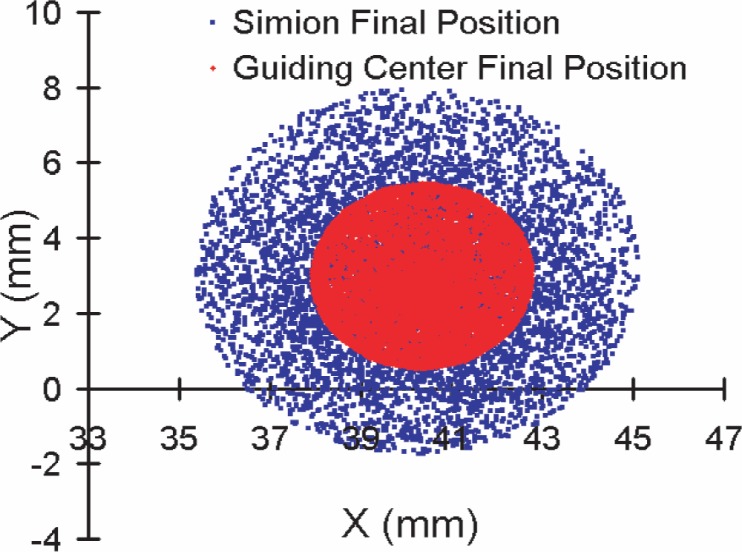
Proton final positions generated by SIMION compared to proton guiding center final positions generated by the custom software.

To independently verify the custom software’s calculations, TOF values and final positions were compared to SIMION results calculated from the same initial conditions and electric and magnetic fields. Figure 6 is the event-by-event TOF comparison, and Fig. 7 is the final position distributions comparison. The TOF values in Fig. 6 agree within ≈ 0.5 %. In Fig. 7, the SIMION final position distribution has a larger radius due to the calculation difference between the full equations of motion and the guiding center approximation, but the important point is that the centers of both distributions overlap.

## 4. Conclusions

Based on the results of the charged particle transport simulations, the spectrometer design chosen for the abBA coincidence experiment guarantees that the initial direction of proton emission is preserved since no “reversed” protons are detected. The preservation of initial proton emission direction is crucial in the determination of the correlation coefficient a. The simulation results also verify that the spectrometer design insures the reliable tracking of charged particles such that the probability distribution of final positions on the Si detector can be determined from initial positions in the neutron decay volume. In addition, the TOF and final position distributions produced by the custom software agree well with those produced by SIMION.

## Footnotes

1 Certain commercial equipment, instruments, or materials are identified in this paper to foster understanding. Such identification does not imply recommendation or endorsement by the National Institute of Standards and Technology, nor does it imply that the materials or equipment identified are necessarily the best available for the purpose.

